# Association between Ranolazine, Ischemic Preconditioning, and Cardioprotection in Patients Undergoing Scheduled Percutaneous Coronary Intervention

**DOI:** 10.3390/medicina60010166

**Published:** 2024-01-16

**Authors:** Konstantinos Kourtis, Angeliki Bourazana, Andrew Xanthopoulos, Spyridon Skoularigkis, Emmanouil Papadakis, Sotirios Patsilinakos, John Skoularigis

**Affiliations:** 1 Department of Cardiology, Konstantopouleio General Hospital, 14233 Athens, Greece; kourtis.konstantinos.md@gmail.com (K.K.); spatsilinakos@gmail.com (S.P.); 2 Department of Cardiology, University Hospital of Larissa, 41110 Larissa, Greecespiros6231998@gmail.com (S.S.)

**Keywords:** ranolazine, preconditioning, cardioprotection, percutaneous, coronary, intervention

## Abstract

*Background and Objectives*: Remote ischemic preconditioning (RIPC) has demonstrated efficacy in protecting against myocardial ischemia–reperfusion injury when applied before percutaneous coronary revascularization. Ranolazine, an anti-ischemic drug, has been utilized to minimize ischemic events in chronic angina patients. However, there is a lack of trials exploring the combined effects of ranolazine pretreatment and RIPC in patients undergoing percutaneous coronary interventions (PCIs). *Materials and Methods:* The present study is a prospective study which enrolled 150 patients scheduled for nonemergent percutaneous coronary revascularization. Three groups were formed: a control group undergoing only PCIs, an RIPC group with RIPC applied to either upper limb before the PCI (preconditioning group), and a group with RIPC before the PCI along with prior ranolazine treatment for stable angina (ranolazine group). Statistical analyses, including ANOVAs and Kruskal–Wallis tests, were conducted, with the Bonferroni correction for type I errors. A repeated-measures ANOVA assessed the changes in serum enzyme levels (SGOT, LDH, CRP, CPK, CK-MB, troponin I) over the follow-up. Statistical significance was set at *p* < 0.05. *Results*: The ranolazine group showed (A) significantly lower troponin I level increases compared to the control group for up to 24 h, (B) significantly lower CPK levels after 4, 10, and 24 h compared to the preconditioning group (*p* = 0.020, *p* = 0.020, and *p* = 0.019, respectively) and significantly lower CPK levels compared to the control group after 10 h (*p* = 0.050), and (C) significantly lower CK-MB levels after 10 h compared to the control group (*p* = 0.050). *Conclusions*: This study suggests that combining RIPC before scheduled coronary procedures with ranolazine pretreatment may be linked to reduced ischemia induction, as evidenced by lower myocardial enzyme levels.

## 1. Introduction

Remote ischemic preconditioning is the phenomenon where one or more episodes of brief ischemia–reperfusion are applied to a distant tissue or organ in order to render the myocardium relatively unsusceptible to the succeeding longer episode of ischemia. While it remains inadequately elucidated, myocardial ischemia–reperfusion injury attenuation is attributed to the upregulation of protective kinases such as protein kinase C and extracellular signal-related kinase in combination with a systemic response involving immune cells’ crosstalk after the initial release of adenosine and bradykinin [1,2,3,4]. In the context of elective and primary percutaneous coronary intervention (PCI) particularly, remote ischemic preconditioning has been shown to confer myocardial protection during PCI due to coronary microvascular function enhancement [5,6].

The precise mechanism underlying ranolazine’s anti-anginal and anti-ischemic effects is still not fully comprehended [7,8]. At therapeutic levels, it hinders the late phase of inward sodium channels in ischemic cardiac myocytes, leading to a decrease in intracellular sodium levels. Consequently, this reduction in intracellular sodium diminishes the entry of calcium through the Na-Ca channel. Lowered intracellular calcium levels result in reduced tension on the ventricular wall, thereby decreasing oxygen demand. Notably, ranolazine has no impact on cardiac workload. When present at elevated levels, it inhibits the rapid delayed rectifier potassium current, causing a delay in the action potential and a prolongation of the QT interval. Furthermore, ranolazine also impedes the oxidation of fatty acids, which, in turn, augments glucose oxidation, limits lactic acid production, and ultimately ameliorates cardiac function [7,8].

To evaluate the possible cardioprotective action of ranolazine in the context of PCI in combination with RIPC, we performed a prospective study to investigate the effect on ischemia as reflected by the myocardial enzyme levels. To the best of our knowledge, this is the first study to assess the potential role of RIPC in patients with chronic angina who were on treatment with ranolazine in the context of PCI.

## 2. Materials and Methods

### 2.1. Patient Population and Study Design

The present study is a prospective, single-center study of patients undergoing scheduled (nonemergent) PCIs. Patients were divided into three groups successively: a control group undergoing only PCI (*n* = 50), an RIPC group with RIPC applied to either upper limb before PCI (preconditioning group, *n* = 50), and a group with RIPC before PCI along with prior ranolazine treatment for stable angina (ranolazine group, *n* = 50).

While being in the catheterization laboratory, a 5 min inflation of a blood pressure arm-sized cuff to 200 mmHg around either of the upper arms, followed by a one-minute deflation of the cuff to 0 mmHg, was performed once in the patients who were assigned to the RIPC group. Right after the one-minute deflation period of the cuff, the catheterization of the radial artery followed. In the patients who were assigned to the ranolazine group, the same procedure regarding the RIPC technique was also followed.

All the patients were submitted to a blood test 4, 10, and 24 h after the coronary intervention, and the levels of C-reactive protein (CRP), creatine phosphokinase (CPK), CK-MB, and troponin I were measured along with a complete blood count and basic renal and liver function measurements (Unicel DxH 600, Beckman USA analyzer, and Dimension EXL, Siemens analyzer, Erlangen, Germany). Additionally, an electrocardiogram (ECG) was performed on all patients before and after their admission in the catheterization laboratory in order to evaluate any evolutionary ST alterations. A cardiac ultrasound (GE, HealthcareVivid e95), evaluating basic echocardiographic parameters, was also performed and reviewed after admission by two independent echocardiographers. All angiographic parameters and quantitative coronary stenosis assessments were recorded. Finally, all patients were provided with the recommendation for exercise abstention before hospital admission in order to avoid any false positive levels of troponin due to cross-reaction because of elevated levels of CPK.

The study was conducted in accordance with the Declaration of Helsinki and approved by the Institutional Review Board (or Ethics Committee) of the University of Thessaly, School of Medicine (protocol code 2563 and date of approval: 7 June 2017). Written informed consent was obtained from all patients before participation.

### 2.2. Outcomes

The aim of the present analysis was to examine and compare the associations between the study groups (control group, RIPC group, and ranolazine group) and the markers of myocardial injury. The study follow-up was up to 24 h after PCI.

### 2.3. Statistical Analysis

Quantitative variables were expressed as mean values (standard deviation) and as medians (interquartile range), while qualitative variables were expressed as absolute and relative frequencies. For the comparison of proportions, chi-square and Fisher’s exact tests were used. An analysis of variance (ANOVA) and the Kruskal–Wallis test were computed for the comparison of mean values. The Bonferroni correction was used in order to control for type I errors. A repeated-measures analysis of variance (ANOVA) was adopted to evaluate the changes observed in SGOT, LDH, CRP, CPK, CK-MB, and troponin I among the different groups over the follow-up period. Log transformations were made in the case of not-normal distributions. A multiple linear regression analysis through a stepwise method (*p* for entry 0.05, *p* for removal 0.10) was used, with the dependent variable being the change in troponin from the pre-operative value to 24 h after. The regression equation included terms for demographics, clinical characteristics, information about participants’ medical history, and the changes in SGOT, LDH, CRP, CPK, and CK-MB. Adjusted regression coefficients (β) with standard errors (SEs) were computed from the results of the linear regression analyses. All reported *p* values are two-tailed. Statistical significance was set at *p* < 0.05, and the analyses were conducted using SPSS statistical software, version 22.0 (Armonk, NY, USA: IBM Corp) and GraphPad Prism 10.1.1 (GraphPad Software, San Diego, CA, USA).

## 3. Results

### 3.1. Baseline Characteristics

The sample consisted of 150 patients aged from 30 to 88 years old (79.3% men), divided into three equally sized groups (50 patients each). Their characteristics are presented in Table 1. Most patents in each group were men and Greek. The mean age was 65.5 years (SD = 11.3 years) for the control group, 66.3 years (SD = 9.7 years) for the preconditioning group, and 66.3 years (SD = 13.4 years) for the Ranolazine group. All the characteristics were similar among the three groups, with the exception of heart rate (Table 1).

### 3.2. Electrocardiography and Echocardiography

The QTc values differed significantly among the three groups (Supplementary Table S1). Specifically, after the Bonferroni correction, it was found that these values in the control group were significantly lower compared to the preconditioning group (*p* = 0.006) and the ranolazine group (*p* = 0.001). Significant differences were found in PWd, IVSs, and AV_Vmax among the three groups. Specifically, after the Bonferroni correction, it was found that the PWd values in the ranolazine group were significantly lower than those of the control and preconditioning groups (*p* = 0.014 and *p* = 0.001, respectively). Also, the values of the IVSs were significantly higher in the preconditioning group compared to the values for the control and the ranolazine groups (*p* = 0.001 and *p* < 0.001, respectively). The AV_Vmax values were significantly higher in the ranolazine group compared to the preconditioning group (*p* < 0.001).

### 3.3. Medical Treatment

After the Bonferroni correction, no differences were found among the three groups in the percentages of IIBIIIA (after PCI) and statins (Table 1). On the contrary, the percentage of nitrates administered during the PCIs was found to be significantly higher in the ranolazine group compared to the control (*p* < 0.001) and the preconditioning groups (*p* < 0.001) (Supplementary Table S2). Also, the percentage of nitrates administered before the PCIs was significantly lower in the ranolazine group compared to the control (*p* < 0.001) and the preconditioning groups (*p* < 0.001). The patients in the preconditioning group received diuretics more frequently than the patients in the control group (*p* = 0.006).

### 3.4. Hematology Markers’ Longitudinal Changes

The changes in SGOT, LDH, CRP, CPK, CK-MB, and troponin I by group are presented in Table 2 and Supplementary Table S3. During the follow-up period, the levels of SGOT changed significantly in all groups (*p* < 0.001) in a similar way (*p* = 0.941). More specifically, in all groups, the SGOT level before the PCI was significantly lower compared to all the post-PCI measurements (*p* < 0.001 for all comparisons).

The levels of LDH changed significantly in all the groups (*p* < 0.001) in a similar way (*p* = 0.340). More specifically, in the control group, the level of LDH was significantly lower before the PCI compared to 4, 10, and 24 h after (*p* < 0.001 for all comparisons). In the preconditioning group, the level of LDH was significantly lower before the PCI compared to 4 and 10 h after (*p* < 0.001 for both comparisons). Also, after 24 h, the LDH levels were significantly lower compared to those after 10 h (*p* < 0.001). In the ranolazine group, the level of LDH was significantly lower before the operation compared to 4 and 10 h after (*p* = 0.001 and *p* < 0.001, respectively). Also, after 24 h, the levels of LDH were significantly lower compared to those after 10 h (*p* = 0.006).

The CRP levels changed significantly in all the groups (*p* < 0.001) in a similar way (*p* = 0.863). More specifically, in the control group, the level of CRP was significantly lower before the PCI compared to 4, 10, and 24 h after (*p* < 0.001 for all comparisons). After 4 h, the CRP level was significantly lower compared to after 10 and 24 h (*p* < 0.001 for all comparisons). Similarly, the CRP level after 10 h was significantly lower compared to 24 h later (*p* < 0.001). In the preconditioning group, the CRP level was significantly lower before the PCI compared to 4, 10, and 24 h after (*p* < 0.001 for all comparisons). After 4 h, the CRP level was significantly lower compared to after 10 and 24 h (*p* = 0.002 and *p* < 0.001, respectively). Similarly, the CRP level after 10 h was significantly lower compared to after 24 h (*p* < 0.001). In the ranolazine group, the CRP level was significantly lower before the PCI compared to 4, 10, and 24 h after (*p* < 0.001 for all comparisons). The amount of CRP after 4 h was significantly lower compared to after 10 and 24 h (*p* < 0.001 for all comparisons). Similarly, the level of CRP after 10 h was significantly lower compared to after 24 h (*p* = 0.002).

The levels of CPK changed significantly in all the groups (*p* < 0.001) in a similar way (*p* = 0.650). More specifically, in the control group, the CPK level was significantly lower before the PCI compared to 4, 10, and 24 h after (*p* < 0.001 for all comparisons). The CPK level after 4 h was significantly lower compared to 10 and 24 h after (*p* < 0.001 and *p* = 0.006, respectively). In the preconditioning group, the CPK level was significantly lower before the PCI compared to 4, 10, and 24 h after (*p* < 0.001 for all comparisons). The level of CPK after 4 h was significantly lower compared to after 10 h (*p* = 0.001). In the ranolazine group, the CPK level was significantly lower before the PCI compared to 4, 10, and 24 h after (*p* < 0.001 for all comparisons). The level of CPK after 4 h was significantly lower compared to after 10 h (*p* = 0.006).

The CK-MB levels changed significantly in all the groups (*p* < 0.001) in a significantly different way (*p* = 0.002). More specifically, in the control group, the CK-MB level was significantly lower before the PCI compared to 4, 10, and 24 h after (*p* < 0.001 for all comparisons). The CK-MB level after 4 h was significantly lower compared to 10 and 24 h after (*p* < 0.001 for both comparisons). The CK-MB values after 10 and 24 h were similar (*p* = 0.870). In the preconditioning group, the CK-MB level was significantly lower before the PCI compared to 4, 10, and 24 h after (*p* < 0.001 for all comparisons). The level of CK-MB after 4 h was significantly lower compared to after 10 and 24 h (*p* < 0.001 for both comparisons). In the ranolazine group, the CK-MB level was significantly lower before the PCI compared to 4, 10, and 24 h after (*p* = 0.001, *p* < 0.001, and *p* < 0.001, respectively). The level of CK-MB after 4 h was significantly lower compared to 10 and 24 h after (*p* = 0.001 and *p* = 0.003, respectively).

**Figure 1:**
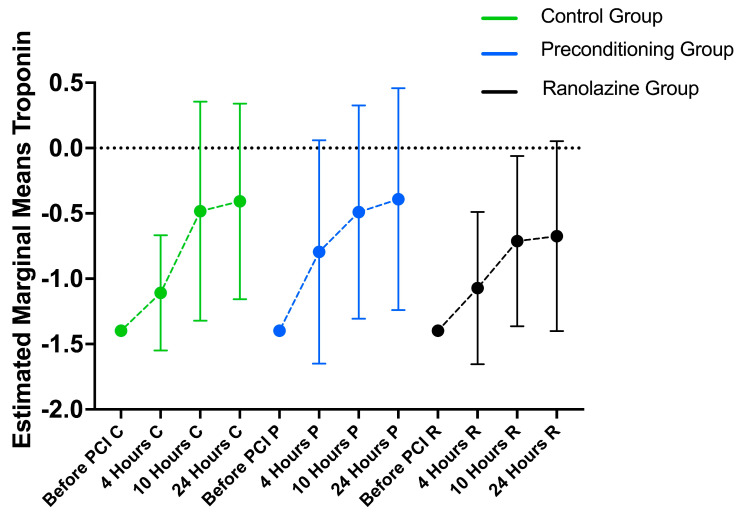
Changes in troponin during follow-up by group. Abbreviations: C, control group; P, preconditioning group; R, ranolazine group.

The amount of troponin I changed significantly in all the groups (*p* < 0.001) in a significantly different way (*p* = 0.040) (Figure 1, Table 2). More specifically, in the control group, the amount of troponin was significantly lower before the PCI compared to 4, 10, and 24 h after (*p* = 0.002, *p* < 0.001, and *p* < 0.001, respectively). The troponin level after 4 h was significantly lower compared to after 10 and 24 h (*p* < 0.001 for both comparisons). In the preconditioning group, the troponin level was significantly lower before the PCI compared to 4, 10, and 24 h after (*p* < 0.001 for all comparisons). The amount of troponin after 4 h was significantly lower compared to after 10 and 24 h (*p* < 0.001 for both comparisons). In the ranolazine group, the level of troponin was significantly lower before the PCI compared to 4, 10, and 24 h after (*p* <0.001 for all comparisons). The troponin level after 4 h was significantly lower compared to 10 and 24 h later (*p* < 0.001 for both comparisons).

### 3.5. Between-Group Differences

**Figure 2:**
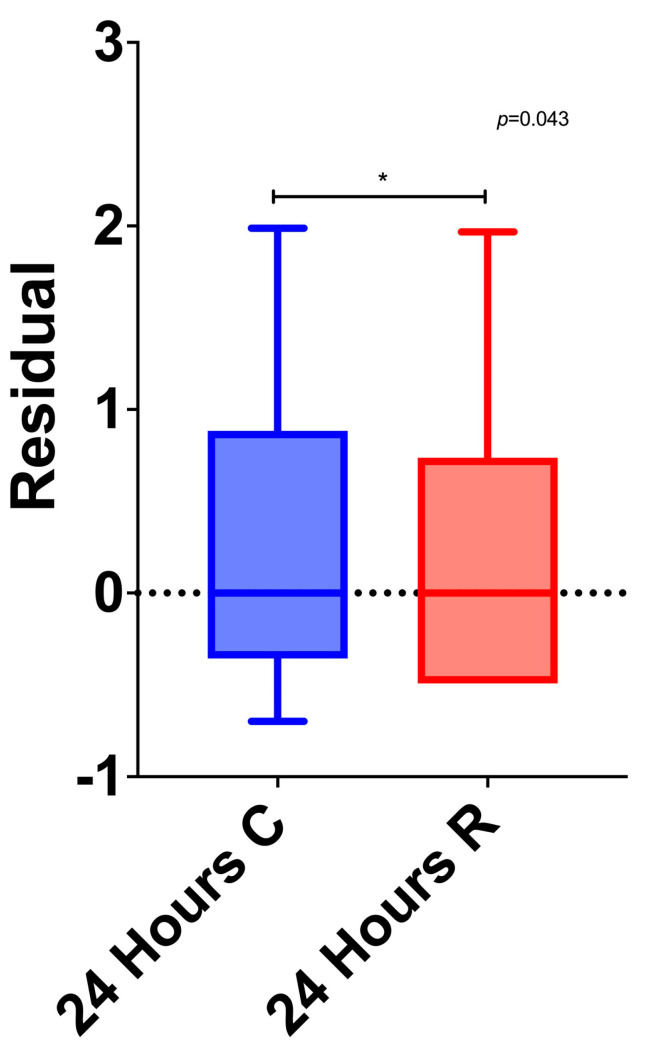
Comparison between ranolazine and control groups’ troponin values after 24 h. Abbreviations: C, control group; R, ranolazine group. * = Significant difference between ranolazine and control groups’ troponin values.

The LDH and CPK levels before the PCI and 4 h after, as well as the CPK and CK-MB levels after 10 h, differed significantly among the three groups. Specifically, after the Bonferroni correction, it was found that initially, the amount of LDH was significantly lower in the control group compared to the ranolazine group (*p* = 0.041). Four hours after the PCI, the amount of LDH was significantly lower in the control group compared to the preconditioning group (*p* = 0.040). The amount of CPK after 4, 10, and 24 h was significantly lower in the ranolazine group compared to the preconditioning group (*p* = 0.020, *p* = 0.020, and *p* = 0.019, respectively). Also, the amount of CPK after 10 h was significantly lower in the ranolazine group compared to the control group (*p* = 0.050). The CK-MB level at 10 h was significantly lower in the ranolazine group compared to the control group (*p* = 0.050). Lastly, the level of troponin was significantly lower in the ranolazine group compared to the control group after 24 h (*p* = 0.043) (Figure 2).

### 3.6. Multivariable Analysis

When multiple linear regression was conducted having the change in troponin as dependent variable it was found that troponin increased significantly less in the Ranolazine group compared to the Control group (Supplementary Table S4). Also, women had significantly greater increase in their troponin compared to men. Greater increase in SGOT and CK-MB values was significantly associated with greater increase in troponin.

## 4. Discussion

RIPC has been proposed to increase myocardial salvage in patients undergoing PCIs in the context of myocardial infarction (MI) with a reduction in the size of the MI and myocardial edema, while ranolazine has been shown to exert a favorable impact on the pathophysiology of periprocedural MI [9,10,11]. We demonstrated that ranolazine administered as a pretreatment to patients undergoing PCIs due to chronic coronary artery disease and to whom RIPC was applied before the intervention exhibited significantly lower levels of myocardial necrosis markers. To the best of our knowledge, this study is the first to provide evidence that ranolazine and RIPC may have a possible synergistic action in alleviating ischemia–reperfusion injury.

Ranolazine administration in combination with RIPC significantly lowered the troponin I and CK-MB levels following the PCIs. These observations are consistent with the benefit of RIPC before PCI that was demonstrated by previous trials and highlight the concept that the positive effects of preconditioning may be amplified by the favorable effects of ranolazine administration [12].

While our understanding of the cardioprotective mechanism of RIPC is still incomplete, preconditioning has been suggested to enhance the stability of susceptible plaques by inhibiting platelet activity and exerting antithrombotic effects [13]. Peri-procedural MI can result from various causes. A recent trial using magnetic resonance imaging has revealed that reduced blood flow in small coronary vessels and the distal release of atherosclerotic material are the main contributors to myocardial tissue damage in the course of PCI. In cases where there are significant elevations in CK-MB or troponin levels, it is more likely that the obstruction of side branches due to the shifting of plaque is the primary contributor. Conversely, when there is only a limited rise in myocardial enzyme levels, it is more likely to be attributed to thrombotic or atheromatous emboli [14,15,16]. In the present work, troponin values, both before and after the PCI, were not different between the preconditioning and the control groups. However, this observation may result from the relatively limited sample size.

In our analysis, we observed a significant elevation in CRP levels after PCI in every study group, which confirms the presence of an inflammatory response during the PCI procedure. However, the CRP levels increased after the PCIs in all the groups in a similar way, suggesting that a containment of the inflammatory response is not the most favorable mechanism for cardioprotection. This is in agreement with previous studies, which similarly did not feature inflammation reduction as the core mechanism of RIPC’s beneficial effect [12].

Understanding the advantageous mechanism by which ranolazine operates in angina pectoris has posed difficulties and has only recently started to become clearer. One potential explanation implicates the concomitant regulation of ion homeostasis. In instances of ischemia, immediate changes occur in the ion homeostasis of cardiomyocytes, including alterations in the intracellular Na^+^, Ca^2+^, and H^+^ levels, as well as the extracellular K^+^ level [17,18,19]. Specifically, an elevation in the amplitude of a persistent or late Na^+^ current (INa,late), observed in various pathological conditions, can extend the duration of the action potential (AP) and raise intracellular Na^+^ levels [20]. The inadequate counterbalance of Na^+^ extrusion through the Na^+^-K^+^ ATPase pump contributes to an accumulation of intracellular Na^+^, leading to an increase in intracellular Ca^2+^. This Ca^2+^ overload raises diastolic left ventricular (LV) pressure, initiating a detrimental feedback loop affecting energy supply and demand, thereby worsening angina pectoris. The heightened intracellular Ca^2+^ level in myocardial cells amplifies the tension on the diastolic wall, resulting in an elevation of end-diastolic pressure. The augmented stiffness of the diastolic wall induces intra-myocardial vascular compression, diminishing the blood flow and oxygen supply to the myocardium and ultimately impeding ventricular filling. The suggested mechanism behind the demonstrated effectiveness and clinical application of ranolazine in treating stress angina involves the blocking of late sodium current (INa,late). By inhibiting INa,late, there is a reduction in the intracellular Na^+^ levels, mitigating Na^+^-induced calcium overload. This, in turn, helps alleviate the adverse effects on the left ventricle’s diastolic pressure, which worsens angina pectoris [21].

Studies so far have assessed the utility of ranolazine administration to patients with acute coronary syndrome who have been submitted to PCI in order to reduce ischemia burden in the long term. The RIVER-PCI failed to demonstrate any significant improvement in ischemia-driven revascularization or ischemia-driven hospitalizations in patients with incomplete revascularization and chronic angina treated with ranolazine as an adjunctive therapy [22]. However, a subgroup analysis of MERLIN-TIMI 36 displayed a significant reduction in the risk of recurrent ischemia and cardiovascular death in patients with chronic angina and PCI in the setting of acute coronary syndrome, treated with ranolazine. Only one study evaluated pre-treatment with ranolazine in patients with stable angina and an imminent scheduled PCI as a means of reducing procedural myocardial injury, demonstrating positive results. However, this was a small-scale study, including 70 patients [5].

The study participants all had stable coronary artery disease and were submitted to percutaneous coronary angioplasty without any significant differences in the number of lesions treated or the number of stents used among the groups. This would set aside the assertion that myocardial enzyme variance is a result of a variation in the extent of periprocedural injury.

### Limitations

The present work was a single-center study, and the number of patients enrolled (n = 150) was not large. The patients were divided into three groups successively (a non-randomized study), and therefore the risk of bias or confounding cannot be excluded. However, it should be regarded as “hypothesis generating”. Furthermore, there was a relatively inhomogeneous sample composition regarding the sex of the patients, with men constituting the majority of the patients in every group, namely 82% in the control group, 78% in the ranolazine group, and 78% in the RIPC group. Despite this, the multivariate regression analysis showed that the females demonstrated a significantly higher troponin value measured at every time point compared with the men.

## 5. Conclusions

The levels of myocardial enzymes after percutaneous coronary intervention in patients with chronic angina are significantly lower when pretreatment with ranolazine and the application of RIPC precede it. The aforementioned hypothesis suggests that ranolazine confers cardioprotection during PCIs when combined with RIPC before an intervention. Nonetheless, it is necessary to validate this observation through large randomized multicenter studies with sufficient statistical power to evaluate significant clinical outcomes.

## Figures and Tables

**Table 1:** Baseline characteristics of the study population.

	Control Group	Preconditioning Group	Ranolazine Group	*p*
	(1) N = 50; 33.3%	(2) N = 50; 33.3%	(3) N = 50; 33.3%	
	N (%)	N (%)	N (%)	
Gender				
Men	41 (82.0)	39 (78.0)	39 (78.0)	0.850 +
Women	9 (18.0)	11 (22.0)	11 (22.0)	
Age, mean (SD)	65.5 (11.3)	66.3 (9.7)	66.3 (13.4)	0.913 ‡
Heart rate, mean (SD)	63.1 (8.5) ^2^	68.5 (9.3) ^1^	65.4 (8.0)	**0.009 ‡**
Systolic blood pressure, mean (SD)	135.8 (8.8)	133.9 (12.8)	133.6 (10.0)	0.553 ‡
Diastolic blood pressure, mean (SD)	79.2 (7.2)	77 (9.0)	78.1 (9.2)	0.440 ‡
Nationality				
Greek	48 (96.0)	45 (90.0)	49 (98.0)	0.277 ++
Other	2 (4.0)	5 (10.0)	1 (2.0)	
Βody mass index, mean (SD)	28.5 (4)	29.3 (4.1)	29.4 (4.8)	0.543 ‡
Βody mass index				
Normal	5 (10.0)	7 (14.0)	10 (20.0)	0.289 +
Overweight	30 (60.0)	21 (42.0)	23 (46.0)	
Obese	15 (30.0)	22 (44.0)	17 (34.0)	
Body surface area, mean (SD)	2.01 (0.17)	1.98 (0.22)	2.02 (0.24)	0.673 ‡
Smoking				
No	17 (34.0)	16 (32.0)	22 (44.0)	0.052 +
Yes, in the past	26 (52.0)	17 (34.0)	14 (28.0)	
Yes, in the present	7 (14.0)	17 (34.0)	14 (28.0)	
Packs/day, median (IQR)	0 (0–1)	0 (0–1)	1 (0–1.3)	0.424 ‡‡
Alcohol consumption	2 (4.0)	1 (2.0)	5 (10.0)	0.277 ++
Glomerular filtration rate, median (IQR)	91.3 (79.1–124.7)	84.8 (67.7–104.2)	90.3 (70.3–110.5)	0.211 ‡‡
Comorbidities				
Hypertention	44 (88.0)	43 (86.0)	44 (88.0)	0.942 +
Dyslipidemia	49 (98.0)	47 (94.0)	43 (86.0)	0.083 ++
Diabetes	24 (48.0)	22 (44.0)	21 (42.0)	0.828 +
Coronary artery disease	33 (67.3)	28 (57.1)	30 (60.0)	0.563 +
Atrial Fibrillation	6 (12.0)	5 (10.0)	4 (8.0)	0.801 +
Chronic obstructive pulmonary disease	2 (4.0)	5 (10.0)	7 (14.3)	0.202 ++
Renal failure	18 (36.0)	10 (20.0)	16 (32.0)	0.188 +
Peripheral vascular disease	7 (14.0)	3 (6.0)	5 (10.0)	0.411 +
Sleep disorder	5 (10.0)	3 (6.0)	6 (12.0)	0.686 ++
Previous stroke/transient ischemic Attack	0 (0.0)	1 (2.0)	3 (6.0)	0.324 ++
Myocardial infarction	27 (54.0)	21 (42.0)	24 (48.0)	0.486 +
Cognitive impairment	1 (2.0)	0 (0.0)	0 (0.0)	0.329 ++
Thyroid dysfuction	8 (16.0)	6 (12.0)	5 (10.0)	0.656 +
Heart failure	11 (22.4)	14 (29.2)	16 (32.7)	0.521 +
PCI characteristics *				
Number of vessels				
1	32 (66.7)	38 (76.0)	31 (62.0)	0.268 +
2	12 (25.0)	12 (24.0)	17 (34.0)	
3	2 (4.2)	0 (0.0)	2 (4.0)	
4	2 (4.2)	0 (0.0)	0 (0.0)	
Number of DESs, median (IQR)	2 (1–3)	1 (1–2)	1 (1–2)	0.420 ‡
Medical treatment *				
Acetylsalicylic acid	50 (100.0)	50 (100.0)	50 (100.0)	-
Clopidogrel	14 (28.0)	12 (24.5)	17 (34.0)	0.572 +
Prasugrel	19 (38.0)	21 (42.0)	11 (22.0)	0.082 +
Ticagrelor	18 (36.0)	17 (34.0)	23 (46.0)	0.418 +
IIBIIIA during PCI	0 (0.0)	2 (4.1)	0 (0.0)	0.107 ++
IIBIIIA after PCI	0 (0.0)	4 (8.0)	0 (0.0)	0.034 ++
Coumarin anticoagulant	2 (4.0)	1 (2.0)	3 (6.0)	0.871 ++
Dabigatran	1 (2.0)	1 (2.0)	0 (0.0)	>0.999 ++
Rivaroxaban	1 (2.0)	1 (2.0)	0 (0.0)	>0.999 ++
Apixaban	2 (4.0)	2 (4.0)	1 (2.0)	>0.999 ++
Statin	50 (100.0)	50 (100.0)	46 (93.9)	0.034 ++
Ezetimibe	5 (10.0)	5 (10.0)	10 (20.4)	0.216 +

+ Pearson’s chi-square test; ++ Fisher’s exact test; ‡ ANOVA; ‡‡ Kruskal–Wallis test; ^1,2^ significant differences after Bonferroni correction. Abbreviations: PCI, percutaneous coronary intervention; DES, Drug Eluting Stent; * Additional data about PCI characteristics and medical treatment are listed in Supplementary Table S2. Bold = Statistical significant.

**Table 2:** Changes in troponin by group.

	Control Group (1)		Preconditioning Group (2)		Ranolizine Group (3)		*p ++*
	Mean (SD)	Median (IQR)	Mean (SD)	Median (IQR)	Mean (SD)	Median (IQR)	
TROPONIN I							
Before PCI	0.04 (0)	0.04 (0.04–0.04)	0.04 (0)	0.04 (0.04–0.04)	0.04 (0)	0.04 (0.04–0.04)	>0.999
4 h	0.16 (0.29)	0.04 (0.04–0.11)	0.46 (1.21)	0.05 (0.04–0.32)	0.5 (1.93)	0.04 (0.04–0.08)	0.202
10 h	1.91 (5.02)	0.19 (0.07–1.13)	1.09 (1.90)	0.21 (0.06–1.18)	0.74 (1.65)	0.13 (0.06–0.42)	0.366
24 h	1. 92 (4.11)	0.27 (0.09–2.04)	2.55 (6.18)	0.33 (0.06–2.1)	0.95 (2.08)	0.13 (0.04–0.68)	0.159
*p +*	<0.001		<0.001		<0.001		

+ *p*-value for time comparisons (after logarithmic transformation); ++ *p*-value for group comparisons (after logarithmic transformation).

## Data Availability

The data presented in this study are available on request from the corresponding author.

## Supplementary Materials

The following supporting information can be downloaded at: https://www.mdpi.com/article/10.3390/medicina60010166/s1, Suppl. Table S1: Patients’ data from electrocardiography, echocardiography and percutaneous coronary intervention; Suppl. Table S2: Patients’ medical treatment; Suppl. Table S3. Changes in SGOT, LDH, CRP, CPK and CK-MB by group; Suppl. Table S4. Multiple linear regression results for changes in troponin in a stepwise method.
