# Supplementary material for: Association between Ranolazine, Ischemic Preconditioning, and Cardioprotection in Patients Undergoing Scheduled Percutaneous Coronary Intervention
Source: Medicina (Kaunas). 2024 Jan 16;60(1):166. doi: 10.3390/medicina60010166 (PMC10820875; doi:10.3390/medicina60010166)
Supplement: Supplementary file 1 [file medicina-60-00166-s001.zip › medicina-2785714-supplementary.pdf]

medicina

Suppl. Material

Association between ranolazine, ischemic preconditioning and cardioprotection in patients undergoing scheduled percutaneous coronary intervention

Suppl. Table S1. Patients' data from electrocardiography, echocardiography and percutaneous coronary intervention

	Control group (1)	Preconditioning group (2)	Ranolizine group (3)	P
	N=50; 33.3%	*N=50; 33.3%*	*N=50; 33.3%*	
	**N (%)**	**N (%)**	**N (%)**	
**Electrocardiography**				
Sinus Rhythm	47 (94.0)	48 (96.0)	48 (96.0)	>0.999+
Atrial Fibrillation	2 (4.0)	2 (4.0)	2 (4.0)	>0.999+
Left Bundle Branch Block	1 (2.0)	5 (10.0)	2 (4.1)	0.226++
QRS Width, mean (SD)	98.8 (19.5)	113.5 (47.2)	101.2 (25.7)	0.062‡
QTc, mean (SD)	401.2 (22.6)[2.3]	419 (36.5)[1]	421.3 (23.0)[1]	**0.001‡**
ST Before PCI				
Normal	49 (98.0)	49 (98.0)	48 (96.0)	>0.999++
Depression	1 (2.0)	1 (2.0)	1 (2.0)	
Elevation	0 (0.0)	0 (0.0)	1 (2.0)	
(-) T waves	0 (0.0)	0 (0.0)	0 (0.0)	
ST after PCI				
Normal	32 (64.0)	38 (76.0)	41 (82.0)	0.061++
Depression	13 (26.0)	9 (18.0)	4 (8.0)	
Elevation	1 (2.0)	3 (6.0)	3 (6.0)	
(-) T waves	4 (8.0)	0 (0.0)	2 (4.0)	
**Echocardiography**				
LVEF, median (IQR)	55 (50 – 60)	55 (45 – 60)	55 (50 – 60)	0.593‡
LVEF>=50	40 (80.0)	32 (64.0)	38 (76.0)	0.170+
LVIDD, median (IQR)	52 (49 – 54)	51 (49 – 54)	50 (48 – 54)	0.350‡
LVIDS, median (IQR)	33 (31 – 37)	35 (31 – 40)	35 (31 – 39)	0.268‡
IVSd, median (IQR)	12 (11 – 13)	11 (10 – 12)	12 (9 – 13)	0.190‡
PWd, median (IQR)	11 (10 – 12)[3]	11 (10 – 12)[3]	10 (9 – 11)[1.2]	**0.002‡**
IVSs, median (IQR)	11 (10 – 12)[2]	12.6 (11.5 – 14)[1.3]	10 (9 – 11)[2]	**<0.001‡**
PWs, median (IQR)	-	18 (16 – 23)	11 (10 – 18)	0.071‡
E/A				

<1	33 (67.3)	32 (66.7)	25 (52.1)	0.218+
>1	16 (32.7)	16 (33.3)	23 (47.9)	
A (m/s), median (IQR)	0.8 (0.7 — 0.9)	0.7 (0.6 — 0.8)	0.8 (0.7 — 0.9)	0.073‡
Ao_root (mm), median (IQR)	32 (30 — 35)	33 (30 — 36)	31 (29 — 34)	0.318‡
AscAo (mm), median (IQR)	35 (32 — 39)	35 (33 — 38)	34 (32 — 36)	0.054‡
IVC (cm), median (IQR)	-	1.4 (1.4 — 1.4)	1.2 (1.2 — 1.2)	0.373‡
LAdiamParast (mm), median (IQR)	-	40.9 (35 — 42.5)	42 (42 — 45)	0.029‡
E/E', median (IQR)	15 (15 — 15)	8.7 (8 — 15)	7.1 (5.2 — 9)	0.360‡
TAPSE, median (IQR)	-	12 (2.1 — 19)	19.5 (19 — 20)	0.139‡
AV_Vmax (m/s), median (IQR)	1.3 (1.3 — 1.5)	1.3 (1.2 — 1.4)[3]	1.5 (1.3 — 1.7)[2]	**<0.001‡**
TR_Vmax(m/s), median (IQR)	-	2 (0.6 — 2.2)	2.2 (2 — 2.9)	0.131‡
PASP (mmHg), median (IQR)	-	24 (18.9 — 29)	19 (15 — 23)	0.217‡
**PCI characteristics**				
Previous PCI	25 (50.0)	21 (42.0)	29 (58.0)	0.278+
Access point				
Left upper limb	9 (18.0)	6 (12.2)	14 (28.0)	0.134+
Right upper limb	41 (82.0)	43 (87.8)	36 (72.0)	
Chest pain during PCI	27 (54.0)	19 (38.0)	25 (50.0)	0.249+
Complications	7 (14.6)	9 (18.0)	8 (16.0)	0.899+

+Pearson’s chi-square test; ++Fisher’s exact test; ‡ANOVA; ‡‡Kruskal-Wallis test
1,2,3 significant differences after Bonferroni correction

Abbreviations: LVEF, Left Ventricular Ejection Fraction; LVIDD, Left Ventricular Internal Diameter in Diastole; LVIDS, Left Ventricular Internal Diameter in Systole; IVSd, Interventricular septum diameter in diastole; IVSs, Interventricular septum diameter in systole; PWd, Posterior wall diameter in diastole; PWs, Posterior wall diameter in systole; Ao_root, Aortic root; AscAo, Ascending aorta; IVC, Inferior Vena Cava; LAdiamParast, Left Atrial diameter parasternal view; PCI, Percutaneous Coronary Intervention; TAPSE, Tricuspid Annular Plane Systolic Excursion; AV, Aortic valve; TR, Tricuspid regurgitation; PASP, Pulmonary Artery Systolic Pressure; DES, Drug Eluting Stent; PCI, Percutaneous Coronary Intervention.

Suppl. Table S2. Patients' medical treatment

	Control group (1)	Preconditioning group (2)	Ranolizine group (3)	P
	N=50; 33.3%	*N=50; 33.3%*	*N=50; 33.3%*	
	**N (%)**	**N (%)**	**N (%)**	
Nitrates during PCI	1 (2.0) [3]	6 (12.0) [3]	48 (96.0) [1.2]	**<0.001+**
Nitrates after PCI	8 (16.0)	7 (14.0)	13 (26.0)	0.256+
Nitrates before PCI	49 (98.0) [3]	50 (100.0) [3]	8 (16.0) [1.2]	**<0.001+**
Ezetimibe	5 (10.0)	5 (10.0)	10 (20.4)	0.216+
Fenofibrate	2 (4.0)	1 (2.0)	1 (2.0)	>0.999++
Calcium Chanel Blockers	10 (20.0)	17 (34.0)	18 (36.7)	0.149+
Ivabradine	2 (4.0)	3 (6.0)	2 (4.1)	>0.999++
Digitalis	0 (0.0)	1 (2.0)	0 (0.0)	>0.999++
Sacubitril/Valsartan	0 (0.0)	1 (2.0)	0 (0.0)	>0.999++
Angiotensin-converting enzyme inhibitors	17 (34.0)	11 (22.0)	13 (26.5)	0.398+
Angiotensin receptor blockers	20 (40.0)	23 (46.0)	26 (53.1)	0.427+
B-Blocker	33 (67.3)	36 (72.0)	41 (83.7)	0.162+
Mineralocorticoid receptor antagonist	2 (4.0)	3 (6.0)	4 (8.2)	0.640++
Diuretics	11 (22.0)[2]	24 (48.0) [1]	19 (38.0)	**0.024+**
Furosemide	4 (8.0)	8 (16.0)	8 (16.0)	0.397+
Hydrochlorothiazide	8 (16.0)	15 (30.0)	13 (26.0)	0.240+
Indapamide	1 (2.0)	1 (2.0)	1 (2.0)	>0.999++

+Pearson's chi-square test; ++Fisher's exact test;
1,2,3 significant differences after Bonferroni correction

Suppl. Table S3. Changes in SGOT, LDH, CRP, CPK and CK-MB by group

	Control group (1)		Preconditioning group (2)		Ranolizine group (3)		P++
	Mean (SD)	Median (IQR)	Mean (SD)	Median (IQR)	Mean (SD)	Median (IQR)	
**SGOT**							
Before PCI	19.4 (4.87)	20 (16 — 22)	20.62 (6.01)	19 (17 — 24)	21.34 (5.79)	20 (17 — 26)	0.270
4 hours	23.06 (6.21)	22 (18 — 26)	25.55 (10.95)	22 (18 — 30)	25.84 (8.78)	23 (20 — 30)	0.304
10 hours	28.72 (15.29)	23.5 (21 — 31)	31.84 (20.39)	24 (20 — 35)	30.46 (10.79)	28.5 (22 — 39)	0.352
24 hours	27.06 (14.48)	22 (19 — 29)	30.59 (19.47)	22 (18 — 34)	28.96 (14.22)	28 (18 — 34)	0.643
P+	<0.001		<0.001		<0.001		
**LDH**							
Before PCI	141.56 (36.05)	138.5 (128 — 158)[3]	157.38 (28.19)	150.5 (139 — 180)	160.52 (29.28)	153 (138 — 177)[1]	**0.027**
4 hours	159.54 (40.59)	156 (134 — 188)[2]	182.64 (43.11)	175 (148 — 207)[1]	176.08 (39.31)	164.5 (149 — 192)	**0.027**
10 hours	173.03 (49.9)	164.5 (138 — 205)	201.49 (114.73)	174 (159 — 215)	191.7 (62.9)	179.5 (160 — 199)	0.084
24 hours	162.54 (43.71)	158.5 (136 — 192)	173.22 (54.32)	160 (142 — 202)	171.1 (31.67)	169.5 (150 — 185)	0.318
P+	<0.001		<0.001		<0.001		
**CRP**							
Before PCI	1.52 (1.24)	1.12 (0.64 — 2.1)	2.15 (2.15)	1.29 (0.67 — 3.2)	1.9 (2.19)	1.3 (0.65 — 2.21)	0.410
4 hours	2.13 (1.64)	1.83 (0.86 — 2.94)	3.07 (3.01)	1.92 (1.14 — 3.81)	2.38 (2.23)	1.62 (1.04 — 2.9)	0.185
10 hours	2.55 (1.59)	2.19 (1.22 — 3.65)	4.14 (4.66)	2.53 (1.77 — 5.58)	3.26 (3.35)	2.31 (1.39 — 3.65)	0.246
24 hours	3.14 (1.9)	2.66 (1.63 — 4.32)	5.5 (7.48)	3.61 (2.39 — 5.04)	3.64 (3.13)	2.95 (1.91 — 3.99)	0.183
P+	<0.001		<0.001		<0.001		
**CPK**							
Before PCI	79.24 (34.69)	81 (51 — 95)	94.38 (58.89)	79 (54 — 107)[3]	69.25 (29.77)	63.5 (52 — 81)[2]	**0.024**
4 hours	99.5 (45.95)	94 (62 — 121)	116.45 (66.84)	102 (71 — 137)[3]	86.47 (48.84)	73.5 (61 — 97)[2]	**0.025**
10 hours	142.08 (105.15)	111.5 (87 — 165)[3]	141.41 (84.97)	117 (85 — 177)[3]	102.81 (61.38)	85.5 (70 — 108)[1.2]	**0.012**
24 hours	130.5 (88.23)	105 (69 — 160)	148.14 (120.4)	108 (81 — 180)	115.12 (123.42)	82.5 (71 — 109)	0.068
P+	<0.001		<0.001		<0.001		
**CK-MB**							
Before PCI	0.93 (3.2)	0.5 (0.2 — 0.7)	0.83 (0.96)	0.6 (0.4 — 0.8)	0.54 (0.73)	0.4 (0.3 — 0.6)	0.133

4 hours	1.53 (2.52)	0.85 (0.5 — 1.4)	1.88 (2.25)	1 (0.7 — 2.4)	1.39 (2.55)	0.8 (0.5 — 0.9)	0.104
10 hours	6.46 (10.99)	1.7 (0.8 — 6.7)[3]	5.7 (11.51)	1.7 (0.9 — 6)	2.59 (4.54)	1.1 (0.8 — 1.9)[1]	**0.047**
24 hours	5.18 (7.93)	1.55 (0.9 — 5.9)	4.91 (8.55)	1.8 (0.9 — 5.9)	3.8 (10.46)	0.95 (0.7 — 2.1)	0.123
P+	<0.001		<0.001		<0.001		

+p-value for time comparisons (after logarithmic transformation)
++p-value for group comparisons (after logarithmic transformation)
1,2,3 significant differences after Bonferroni correction
Abbreviations: SGOT, serum glutamic-oxaloacetic transaminase; LDH, Lactate Dehydrogenase; CRP, C reactive Protein; CPK, Creatinine Phosphokinase.

Suppl. Table S4. Multiple linear regression results for changes in troponin in a stepwise method

		β+	SE++	P
Group	Control (reference)			
	Preconditioning	-0.008	0.038	0.839
	Ranolazine	-0.073	0.037	**0.050**
Gender	Men (reference)			
	Women	0.142	0.038	**<0,001**
Change SGOT (from pre-PCI until 24 hours)		0.009	0.002	**<0.001**
Change CK-MB (from pre-PCI until 24 hours)		0.013	0.003	**<0.001**

Note. Regression was conducted after logarithmic transformation of the dependent variable

+regression coefficient ++Standard Error
